# Evidence-based recommendations for shower-out practices: assessing virus removal from hair using a surrogate non-enveloped virus

**DOI:** 10.1128/spectrum.00387-26

**Published:** 2026-07-13

**Authors:** Anastasia Accoti, Maria N. B. Cajimat, Catherine Olal, Krystle Hensley, Joseph Kozlovac, Dennis A. Bente

**Affiliations:** 1 University of Texas Medical Branch, Galveston National Laboratory and Department of Microbiology and Immunology 547647, Galveston, USA; 2 United States Department of Agriculture, Agricultural Research Service, National Bio and Agro-Defense Facility and Office of National Programs Animal Production and Protection, Washington, DC, USA; NHLS Tygerberg/Stellenbosch University, Cape Town, South Africa; Central Veterinary Institute, Lelystad, Flevoland, Netherlands; University of Wisconsin-Madison, Madison, Wisconsin, USA

**Keywords:** non-enveloped-virus, foot and mouth disease virus, highly-containment laboratories, biosafety shower-out practices, surrogate virus Bovine rhinitis B virus

## Abstract

This study aims to assess the risk of fomite transmission of non-enveloped viruses from different hairstyles and hair types by evaluating shower-out practices used at various biocontainment levels. An example of a non-enveloped virus is Foot and Mouth Disease Virus (FMDV), a contagious pathogen of livestock. Due to its infectivity and environmental stability, outbreaks can lead to catastrophic economic losses. To conduct the study at a lower biocontainment level, we used Bovine Rhinitis B virus (BRBV), a genetically closely related surrogate for FMDV. First, we assessed the inactivation capacity of shampoos and leave-in conditioners directly against BRBV and an enveloped virus for comparison using liquid suspension assay. We show that only one shampoo inactivated BRBV; however, similar to disinfectant agents, all shampoos tested inactivated the enveloped virus. Co-wash and leave-in conditioner products showed neither antiviral activity against BRBV nor the enveloped virus. Second, we investigated the mechanical removal of BRBV on hairs using four different hair products and water, mimicking a personal shower. We used mannequin heads styled with various hairstyles, made of synthetic or human hair. A 3-min active shampoo massage followed by a 2-min water rinse was performed for all hairstyles and hair types. We found that water alone removed BRBV particles from most hairstyles and hair types. Exceptions were observed for one treatment using a synthetic voluminous curly/kinky hairstyle and two treatments involving mannequin head equipped with 100 synthetic braids. This study enhances our understanding of the critical role of personal protective measures in biocontainment facilities. It underscores the necessity of a minimum 2-min shower after exposure to non-enveloped animal viruses such as BRBV and highlights the need to develop specific shower procedures tailored to different hairstyles and hair types.

**IMPORTANCE:**

Shower-out procedures are a critical yet understudied component of biocontainment practices, particularly for preventing fomite transmission of highly stable, non-enveloped viruses. This study provides the first experimental, evidence-based evaluation of virus removal from hair using a surrogate for Foot and Mouth Disease Virus, addressing a significant gap in biosafety knowledge. By systematically assessing the roles of hair products, water rinsing, hairstyles, and hair types, we demonstrate the mechanical removal through rinsing, rather than chemical inactivation by shampoos, is the primary determinant of viral decontamination for most hairstyles. Importantly, our findings identify hair volume and density as key factors for viral persistence, especially in high-volume synthetic hairstyles. Additionally, the observed residual virus following co-wash treatment suggests that oil-rich, low surfactant hair products may impede mechanical virus removal and represent an overlooked risk factor in shower-out practices for high-containment personnel.

## INTRODUCTION

In the United States, there are more than 100 high-containment biosafety research facilities, including Biosafety level 3 and Biosafety level 4 (BSL3 and BSL4) dedicated to studying infectious diseases in humans and animals, particularly those transmitted via air and those with no treatment(s) or effective vaccine(s) (1). There is no international body that monitors or regulates high-containment lab research; however, these facilities follow comprehensive guidelines on design, operation, and safety procedures set by the Biosafety in Microbiological and Biomedical Laboratories (BMBL), a manual developed and published by the US Department of Health and Human Services (2). Work with pathogens that pose high risk to crops and livestock designated as high-consequence foreign animal and pests by the US Department of Agriculture (USDA) and Animal and Plant Health Inspection Service (APHIS) is conducted in Animal Biosafety Level 3-Agriculture (ABSL-3Ag) facilities (2). Animal BSL-3Ag facilities are specialized research environments designed to safely study pathogens of crops and livestock, incorporating enhanced containment features to prevent accidental release of pathogenic agents. ABSL-3Ag facilities are designed for large and loose-housed animals, with the laboratory building acting as the primary barrier between infectious agents and the environment. An illustrative example of work requiring ABSL-3Ag containment is research on Foot and Mouth Disease (FMD), a viral infection recognized as an economically significant disease of domestic livestock (3, 4). FMDV belongs to Picornaviridae family (genus *Aphthovirus*) and can infect a wide range of cloven-hoofed animals, including both ruminants and suids, some of which are of agricultural importance in the United States (5). FMDV is highly stable and remains infectious in the environment outside of animal hosts (6). The virus’s stability in animal secretions (droplets), its persistence on surfaces of farm and laboratory equipment, and fomites have been studied extensively (7–9). FMDV can persist for different lengths of time in animal hide and wool depending on humidity and temperature of storage conditions (10, 11). FMDV can also be recovered from contaminated clothing and footwear, including personal protective equipment (boots, coveralls, gloves) used in laboratories and farms (12, 13). Furthermore, there is evidence that FMDV can survive 24–48 h in the nasal cavity of persons handling infected animals (14). Finally, there is experimental evidence that demonstrates humans can serve as mechanical vectors of FMDV, transmitting the virus to susceptible animals through contaminated fomites such as clothing or footwear (12, 13). Studies have shown that implementing basic hygiene measures, including changing clothing and footwear, thorough handwashing, and showering, significantly reduces and, in some cases, eliminates the risk of transmission to other animals as well as to the outside environment (10, 13, 14).

Human hair could also be a potential source of viral contamination; however, only a few studies have examined human hair as a fomite. One was conducted in the 1960s, and in this study by Barbeito and others, the authors tested the contact transmission from a contaminated beard on a mannequin to a suitable host with both Newcastle disease virus and *Clostridium botulinum* toxin, type A. The results showed that beards retained microorganisms and toxins despite washing with soap and water (15). The other study on the role of hair as fomite was conducted in farm settings, where workers wore boots and coveralls, with or without head coverings. Samples were collected from these materials by swabbing, including hands and human hair, and finding Aleutian mink disease virus and Porcine circovirus-2 in hair samples from people who did not wear head coverings (16).

As the number of biocontainment laboratories has increased and the diversity of the personnels has changed, increased concerns regarding the removal of viral contaminants from different hairstyles and hair types have been raised. No or minimal evidence-based risk assessment has yet established clear criteria or guidelines on how different hairstyles and hair types affect pathogen transmission. Likewise, no specific recommendations exist regarding the use or suitability of hair products to support effective shower-out practices in high-containment facilities (17).

Given this critical gap in applied biosafety research, targeted studies are urgently needed to generate scientific evidence that can support risk-based, harmonized decontamination practices, particularly for shower-out procedures.

To address this issue, the present study investigates the potential risk of fomite transmission of FMDV across different hairstyles and types using BRBV, a known BSL-2 surrogate with genetic, and functional properties similar to other *Aphthoviruses* (18, 19). Specifically, first, we tested the inactivation ability of different hair products against BRBV and an enveloped virus as a comparison, using a liquid suspension assay. Second, we assessed the mechanical removal of the non-enveloped virus, BRBV, from different hairstyles and hair types using four hair products tested before.

## RESULTS

### Liquid suspension assay of non-enveloped and enveloped viral inactivation by hair products

To evaluate viral inactivation due to direct contact with various hair products, a panel comprising four different shampoo brands, one co-wash, and three leave-in conditioners was used. The hair product selection was based on BSL-4 lab users’ preference, facility recommendations, and/or products used at Plum Island Animal Disease Center (PIADC). Full details on the hair products are available in Table S1, and a representative figure of the hair products used is shown in Fig. S1. Two viruses were used: BRBV, a non-enveloped virus used as a BSL-2 model for FMDV, and Hazara Virus (HAZV) (20), a surrogate enveloped virus for Crimean-Congo Hemorrhagic Fever (CCHF). Each hair product was diluted 1:10 in deionized water and then incubated for 3 min with the viral suspension at a ratio of 1:1. The 3 min exposure time was picked to mimic the time duration of body/hair washing used in standard shower-out practices (Plum Island Animal Disease Center standard operating procedures and UTMB Galveston National Laboratory and Robert E. Shope, MD BSL4/ABSL4 Laboratory).

**Fig 1:**
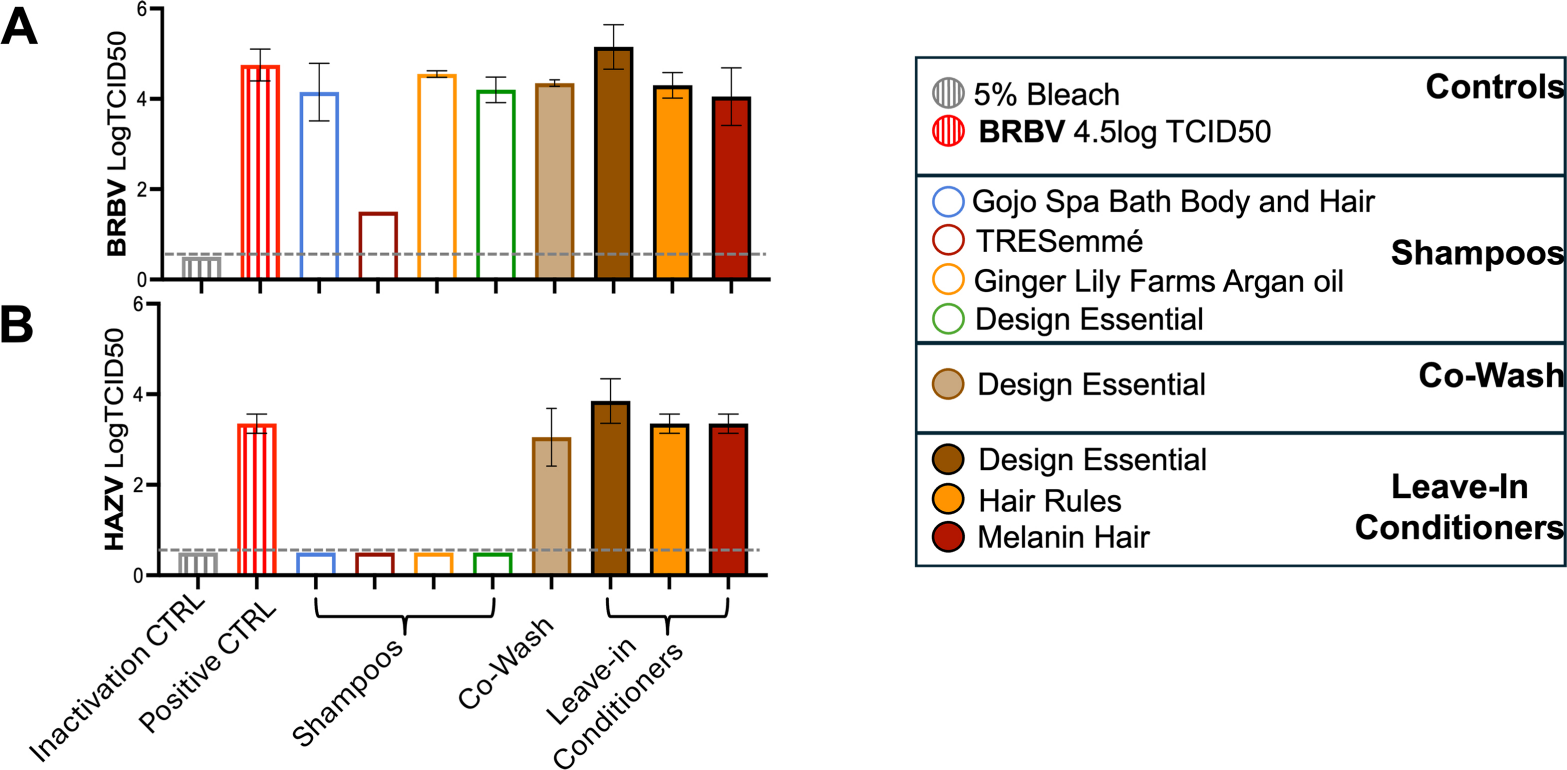
Liquid suspension assay of non-enveloped and enveloped viral inactivation by hair products. The figure shows the results of the liquid suspension assay of non-enveloped. (**A**) and enveloped (**B**) viral inactivation by hair products. The viral titer is expressed as log₁₀ TCID50 of BRBV (**A**) and HAZV (**B**) and was determined using the endpoint dilution method. To assess viral titers, each virus was inoculated onto goat tongue cells (for BRBV) or SW-13 cells (for HAZV) after a 3-min direct treatment with a liquid suspension of various hair products (diluted 1:10 in deionized water). Each hair product was mixed in a 1:1 ratio with 5 × 10^5^ TCID50 of HAZV or BRBV or media, as a cytotoxic control, and serially diluted 10-fold in EMEM + 5% FBS from 10⁻^1^ to 10⁻^6^. The treated virus suspensions were then inoculated into six wells of goat tongue or SW-13 cells and incubated at 33°C (for BRBV) or 37°C (for HAZV) for 7 days. After incubation, cells were fixed and stained with 1% crystal violet in 10% formalin for 1 h. Viral titers (TCID50) were calculated using the Reed and Muench method. Two independent biological replicates were performed. Inactivation CTRL – 5% Bleach; Positive CTRL virus in MEM + 5% FBS. Shampoos: GOJO (1), TRESemmé (2), Ginger (3), Design Essentials, Natural Almond & Avocado, DE (4); Co-wash: Design Essentials, DE (5); Leave-In/Conditioner: Design Essentials Almond & Avocado Detangling, DE (6), Hair Rules Nourishment (7), Melanin Multi-use softening (8).

In order to be considered a total inactivation agent, the hair product must inactivate (i.e., reduce the titer) the virus >4 log_10_(21, 22). Following 3 min of direct contact exposure of BRBV with different hair products, only one shampoo, TRESemmé, demonstrated partial inactivation of BRBV, reducing viral titers by 3.2 log_10_. In contrast, the other three shampoos, the co-wash, and the leave-in conditioners exhibited minimal anti-viral activity against BRBV, with reductions less than 1 log_10_. A 5% bleach solution used as an inactivation control showed a 4.5 log_10_ decrease in BRBV titer. A positive control using media containing 4.5 log_10_ TCID50 BRBV was used. The TCID50 of BRBV across two independent biological replicates is shown in Fig. 1A.

The enveloped virus HAZV, used as comparison, was inactivated by all four shampoos used, showing inactivation levels comparable to the 5% bleach control (a 4.5 log_10_ decrease). The co-wash product showed minimal inactivation activity (1.7 log_10_ reduction), while the leave-in conditioners exhibited minimal or absent inactivation property, with reductions ranging from 0.9 to 1.7 log_10_. The TCID50 results for HAZV across two independent biological replicates are displayed in Fig. 1B. For every liquid suspension assay against the non-enveloped (BRBV) and enveloped (HAZV) viruses, a parallel experiment with only media and hair products was performed to identify the cell cytopathic effects (CPEs) created by the natural cytotoxicity of the hair products.

### Evaluation of titration of bovine rhinitis B virus in goat tongue cells

**Fig 2:**
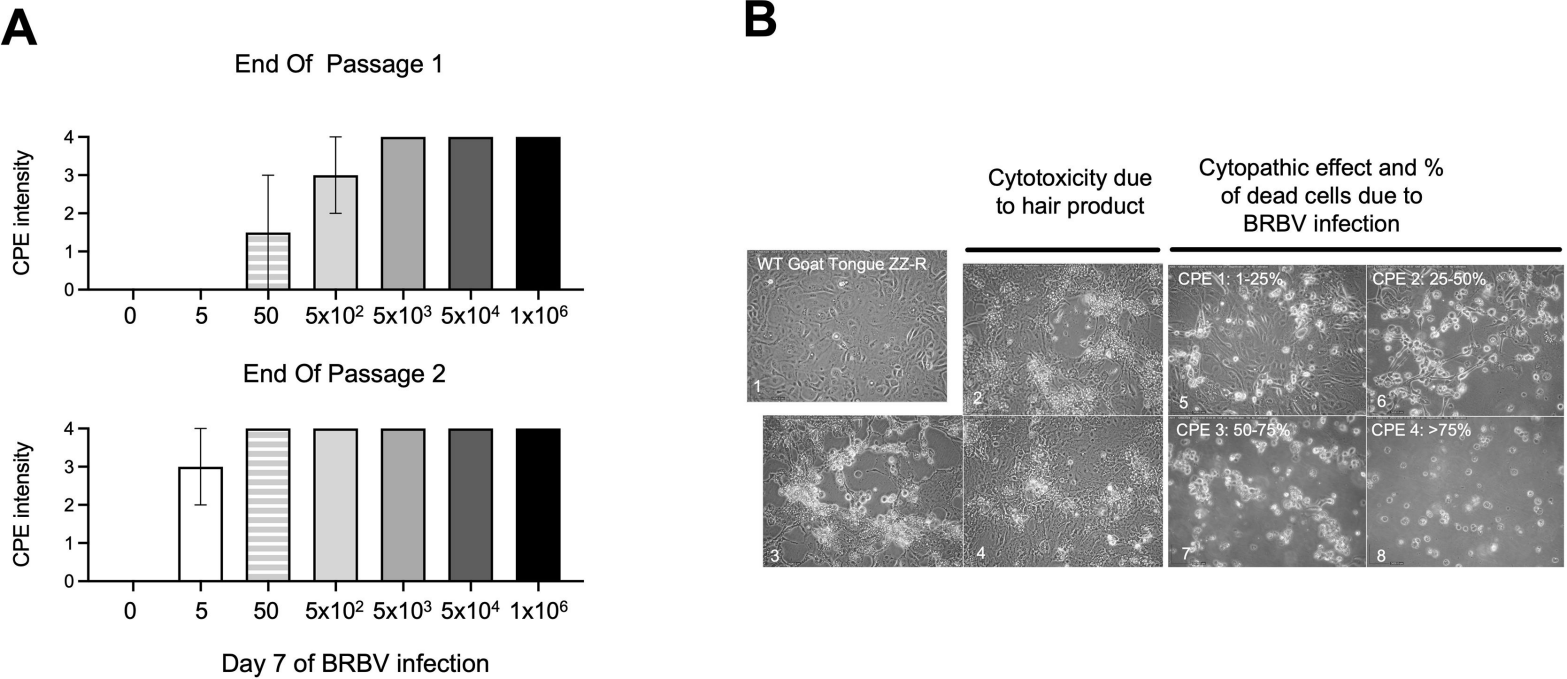
Evaluation of titration of BRBV in goat tongue cells. Panel **A** shows the limit detection for BRBV recovered from hairs. Synthetic Curly/Kinky hair was used to assess the cellular limit of detection for BRBV recovered from two hair loops, each 6 inches long. A 5.3 log TCID50/mL virus solution was serially diluted in media (EMEM, 5% FBS, 1% anti/anti) to 10^−5^ in a final volume of 1 mL. The hair loops were placed in a plastic reservoir and exposed to 1 mL of each dilution in two replicates. The hair loops exposed to virus were left to dry for 1 h, and then, the viral particles were harvested, shaken, and incubated for 5 min followed by filtration through a 0.2 µM filter. The recovered virus was incubated with the cells for 1 h. Infection was assessed over two passages, each lasting 7 days, by monitoring CPE in goat cells. A CPE scoring system (1–4) was used to quantify infection levels: CPE 1: 1%–25%, CPE 2: >25%–50%, CPE 3: >50%–75%, CPE 4: >75. Two biological replicates were performed. Pnael **B** shows goat tongue cells representative images either not infected (1), different grade of cytotoxicity effect due to shampoo treatment or hair itself (2: mild, 3: harsh, 4: extremely harsh) or infected with BRBV displaying progressive characteristic cytopathic effect described above (5: CPE1, 6: CPE 2, 7: CPE 3, 8: CPE 4).

Here, we establish a sensitive infection model using BRBV as a surrogate for FMDV and goat tongue epithelial cells (ZZ-R from Friedrich Loeffler Institute, Germany). Despite being a bovine virus, Bovine Rhinitis B virus did not show CPE in Madin Darby Bovine Kidney (MDBK) cell line and lost CPE upon continuous passage in primary bovine kidney cell culture (19, 23). Previously, this cell type was used to grow FMDV (24). Since BRBV did not produce detectable CPE in MDBK and only RNA was detected by RT-qPCR or by Probe assays (25, 26), we sought to determine if BRBV can infect goat tongue cells and produce CPE similar to FMDV. BRBV inoculation of goat tongue cellsincubated at 33°C resulted in rapid infection and detectable CPE in 48–72 h (Fig. 2B) with a concomitant drop in Ct value as measured by RT-qPCR (data not shown). The highest titer by the TCID50 method coincided with the lowest Ct value.

### Minimum amount of non-enveloped viral particles detected on synthetic hair

To determine the minimum detectable amount of BRBV in synthetic hair, goat cells were infected with five serial dilutions of the virus, and infection was monitored over two 7-day passages. At the end of the first passage, the minimum detectable titer was 50 TCID50. Interestingly, after the second passage, even the lowest concentration tested (5 TCID50) yielded a positive result, confirming the high sensitivity of our model (Fig. 2A).

As mentioned, BRBV infection in goat cells demonstrated high specificity, with distinct cytopathic effects evident even at early stages of infection (Fig. 2B). In the early phase, when less than 25% of cells are dead, infection produces circular zones of dead cells that appear round and translucent. As the disease progresses and cell death reaches approximately 50%, the monolayer takes on a web-like appearance: cells lose their structural integrity, begin to clump, and detach (Fig. 2B). Importantly, the cytopathic effects induced by BRBV infection are clearly distinguishable from the cytotoxic effects caused by hair products when present (Fig. 2B).

### Design of shower-out simulation in a biosafety cabinet for the mechanical removal of non-enveloped virus from hair

**Fig 3:**
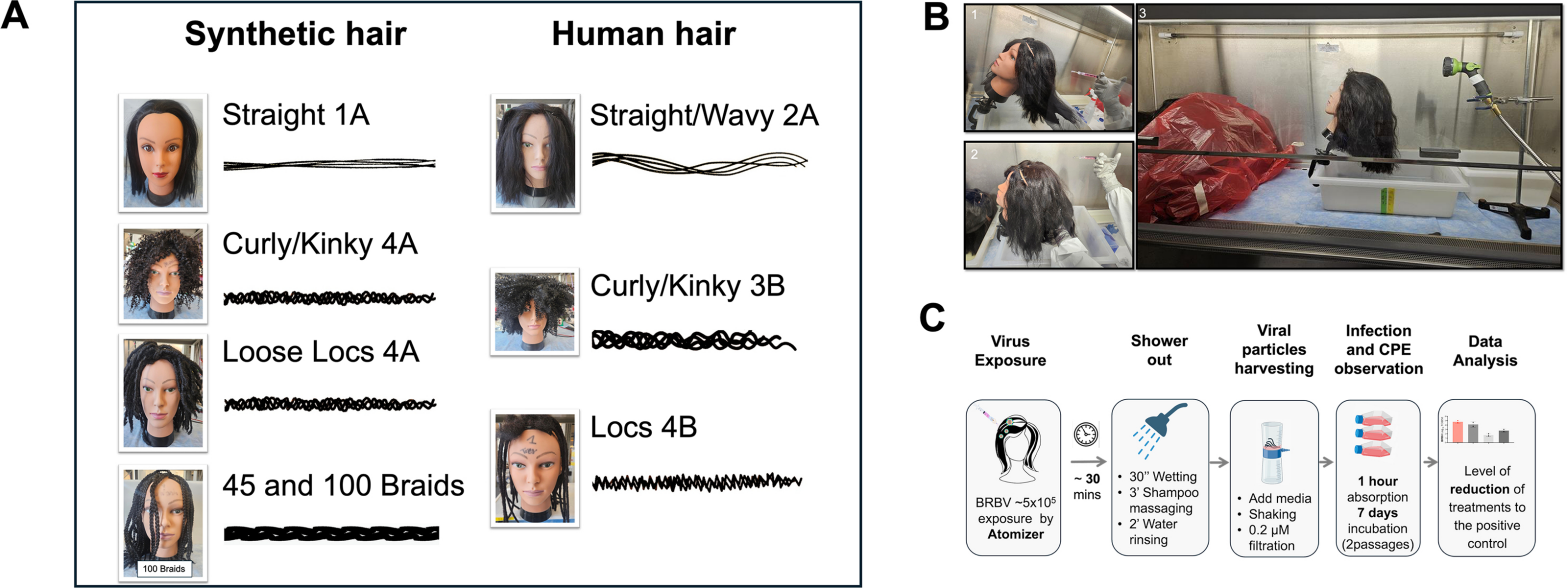
Mechanical removal of BRBV from different hair styles and types by showering in a biosafety cabinet. Panel **A** shows a representation of the hairstyles and types used for the mechanical removal of the FMD surrogate virus, BRBV, during shower-out procedure. The hair styles were defined using, as support, the Andrew Walker hair typing system. The system is split into four main styles (1–4) with substyles labeled A, B, and C. The synthetic hair has the following hair styles, Straight 1A, Curly/Kinky 4A, Loose Locs 4A, 45 and 100 braids. The human hair has Straight/Wavy 2A, Curly/Kinky 3B, Locs 4B. Panel **B** shows the biosafety cabinet showering system, (1) shows the mannequin head anchored through a hook in an autoclave bin and (2) shows BRBV exposure using an atomizer. (3) shows the complete set-up with the first mannequin head ready to be showered and the other mannequin heads covered by a hazard bag to protect them from the shower water. Panel **C** shows the work-flow of the mechanical removal of BRBV during showering; briefly, the mannequin heads were first exposed to ~5 × 10^5^ BRBV viral particles and left to dry for ~30 min. Each head was then massaged for 3 min with specific hair product and then rinsed with water for 2 min. To confirm the mechanical removal of the viral particles from the hair after showering, three representative sections, front, center, and back, of the head were chosen to be collected and used to harvest the eventual viruses. Each hair strand was independently processed by shaking for 5 min and filtered with a 0.2 μM filter. The resulting inoculum was used to infect goat cells for 1 h. Infection was monitored over two passages of 1 week each. Data were analysed qualitative by the presence or absence of infection.

To test the mechanical removal of BRBV from hair, a shower setup was created under BSL-2 conditions (Fig. 3B). A shower hose was attached to the water system and hung within the biosafety cabinet on a lab glassware metal support stand. A standard flow of 4–5 liters/minute was chosen (27). For viral exposure, to best mimic animal droplets, an atomizer was chosen, and due to its ability to fine-aerosolize the liquid, in contrast to pipetting or spraying, all the viral suspension dissolved and dried on the hair within 30 min. To perform the shower of the hairs, mannequin heads, already equipped with a hook, were secured in a 20-liter autoclave bin to ensure full access to the whole head. After viral exposure, each head was secured individually to the short side of the autoclave bin and remained in place for the entire duration of the massage and washing procedure. This setup allowed all shower water containing viral particles and hair products to drain into the autoclave bin, pre-labeled with tape, to measure volume of waste water. After completion of the hair sampling, the liquid volume was measured and bleach was added to obtain a final concentration of 5% and then incubated for 3 min to inactivate any viral particles.

To test different hairstyles and types, initially, cosmetology mannequin heads with pre-attached hairs were used. Unfortunately, the hair was extremely cytotoxic to the cells, leading to total cell death (Fig. 2B). To overcome this issue, a bald mannequin head was equipped each time with pre-tested hair (for cytotoxicity). Different hairstyles and types were chosen to provide a comprehensive collection of hairs. Moreover, to provide an adequate sample collection size sufficient to demonstrate the presence of the virus, three different sections of the mannequin head hair were collected from the front, center, and back of the scalp, and the hairstyle and hair type used are displayed in Fig. 3A. Finally, once every hair strand was collected, the rest of the hair was discarded in a hazard bag. To decontaminate the bald mannequin heads, each head was submerged for 3 min in the shower waste water supplemented with 5% bleach. Pictures of the set-up of biosafety cabinet used to shower mannequin heads and a graphic workflow of the shower-out experiment are displayed in Fig. 3B and C, respectively.

### Evaluation of the mechanical removal of non-enveloped virus from different styles of synthetic and human hairs by showering

To test if different hairstyles (straight/wavy, curly/kinky, braided, and twisted in loc hairs) and different hair types (synthetic and human) resulted in differences in the mechanical removal of BRBV particles from the hair during shower-out practice, mannequin heads equipped with different hairstyles were exposed to 5 × 10^4^ to 3.1 × 10^6^ TCID50 BRBV for 30 min to 1 h before showering. For details on the hair types and brands used, pre-showering conditions, and massaging techniques, refer to Table S2. The mannequin heads with the hair were washed with a 1:5 liquid suspension of three different shampoos and one co-wash previously tested in the liquid suspension assay. A mechanical removal control, called “water rinse,” where only water was used to wash the mannequin head, and a positive virus exposure control, where mannequin heads were exposed to the viral particles and not rinsed with water, were also used. All the synthetic hair tested negative after being washed with the four hair products or rinsed with water, except for two. One of the synthetic curly/kinky hair strands collected from the front, washed with the co-wash hair product, and resulted in a positive result at the end of passage 1. The 100 synthetic braids tested positive in multiple hair collections. The front of the hair washed with TRESemmé showed CPE at the beginning of P2, and the front and back hair strands collected from hair washed only with water tested positive during passage 1. All the human hairs tested were negative after being washed with the four hair products or rinsed with water only. A summary table showing the results of the infection at the end of passage 2 is shown in Table 1, together with the BRBV titer used to expose each hair type.

**TABLE 1:** Summary of passage 2 result of mechanical removal of non-enveloped virus from synthetic and human hairs by showering^*d*^

Hair treatment	Synthetic hair					Human hair		
	Straight	Kinky	Loose Locs	45 Braids	100 Braids	Straight/ Wavy	Kinky	Locs
Ginger- Shampoo	0/3	0/3	0/3	0/3	0/3	0/3	0/3	0/3
TRESemmé- Shampoo	0/3	0/3	0/3	0/3	1/3^*a*^	0/3	0/3	0/3
De Essentials- Shampoo	0/3	0/3	0/3	0/3	0/3	0/3	0/3	0/3
De Essentials- Co-wash	0/3	1/3^*a*^	0/3	0/3	0/3	0/3	0/3	0/3
Water	0/3	0/3	0/3	0/3	2/3^*b*^	0/3	0/3	0/3
Bovine Rhinitis B Virus**^*c*^**	1.4 × 10^6^	3.1 × 10^5^	1.8 × 10^5^	3.1 × 10^6^	1.4 × 10^6^	4 × 10^5^	3.1 × 10^5^	5.5 × 10^4^

^*a*^ Means that the front hair strands resulted positive.

^*b*^ Means that the front and the back hair strands resulted positive.

^*c*^ Titer of the inoculum used to exposed the head, left at room temperature for the same amount of time of the BRBV-mannequin head exposure.

^*d*^ The table is showing the end of passage 2 of the viral infection result of the mechanical removal of the FMD surrogate virus, BRBV, during shower-out practice, and the titer used to expose each hair style. Bald mannequin heads were fitted different hairstyles of synthetic (Straight, Curly/Kinky, Loose Locs, 45 and 100 braids) or human hairs (Straight/Wavy, Curly/Kinky, Locs). Inside a biosafety cabinet, six mannequin heads per each hairstyle were exposed to 5 × 10^4^ to 3.1 × 10^6^ TCID50 and then let dry for 30–60 minutes. The hairs were first wet for 30 s and then massaged with three different shampoos and one co-wash for 3 min, followed by rinsing with water for 2 min. As a mechanical removal control, mannequin heads were rinsed with water for 2 min and 30 s without shampoo. Additionally, not shown in the table, for further control, hair exposed to the virus but no water rinse (viral exposure control) and hair not exposed to the virus (cytotoxicity control) were used. Positive and negative flasks were also used but are not shown in the table. After shampooing and rinsing, hair samples were collected by cutting from the front, center, and back of the mannequin head. The samples were then shaken and incubated for 5 min in EMEM + 5% FBS to recover residual virus, followed by filtration through a 0.2 µM filter. The recovered virus was incubated with the cells for 1 h. Infection was assessed over two passages, each lasting 7 days, by monitoring cytopathic effect (CPE) in goat cells. A CPE scoring system (1–4) was used to quantify infection levels: CPE 1: 1%–25%, CPE 2: >25%–50%, CPE 3: >50%–75%, CPE 4: >75%. Culture medium from the end of Passage 2 (P2) and the initial inoculum (P0) were titrated using an endpoint assay to confirm that CPE was due to BRBV infection.

All the hairs exposed to the virus and not rinsed with water, used as a viral exposure control, yielded positive results during passage 1 and/or 2 except for the hair strand collected from the back of the synthetic straight hairs, the back of the natural curly/kinky hairs, and the back of the human locs. The back titration of the viral exposure control harvested on the day 0 of the experiment revealed a range of viral load per strand from less than 10 to 3.1 × 10^3^. Mild or no cytotoxic effect was detected in the cytotoxicity hair control. The BRBV titer recovered from the three hair strands used as an exposure control—no rinse and the volume of virus used to expose them is listed in Table S3. A detailed version of the results of the three hair strands collected at the end of passage 1 and at the end of passage 2 is shown in Table S4 and Table S5, respectively. The back-titration of the end of P2 samples showed positive results, consistent with the data of the observed CPE during the two passages.

## DISCUSSION

This study is the first to evaluate the efficacy of the shower-out procedure in the removal of FMDV from personnel hair. FMDV is a contagious pathogen of livestock, and an outbreak can result in severe economic consequences and catastrophic financial losses. This study was designed first to assess the effectiveness of commonly used hair products in inactivating non-enveloped viruses in comparison with enveloped viruses, using a liquid suspension assay. The assay used BSL-2 virus surrogates, BRBV was used as a surrogate for FMDV, representing non-enveloped virus, while HAZV was used as a surrogate for CCHFV, representing the enveloped virus. Notably, when tested against the non-enveloped virus BRBV, only one shampoo (TRESemmé) demonstrated mild antiviral activity, reducing viral titers by more than a 3 log_10_-fold. As outlined in Table S1, this formulation contains a relatively high concentration of anionic surfactants and maintains an acidic pH, both of which are known to destabilize non-enveloped viral capsids (28). It is known that FMD viral particles will lose infectivity at pH slightly below neutral, as well as the equine rhinitis A virus (ERAV), a respiratory virus of equids, another closely related member of the *Aphthovirus* genus (29, 30). The pH sensitivity and other physical and chemical attributes of BRBV have yet to be studied; however, it has been demonstrated for a number of non-enveloped viruses that the combination of sodium dodecyl sulfate, SDS (an anionic surfactant), and acids effectively inactivated the viruses, compared to treatment with SDS or acid alone (31, 32).

By contrast, the other shampoos, co-wash, and leave-in conditioners lacked these key components, which may explain their minimal efficacy against BRBV. As expected, HAZV, the enveloped virus, was uniformly inactivated by all shampoos tested. This observation aligns with the lipid envelope’s intrinsic fragility, which is readily disrupted by surfactants, detergents, and even mechanical agitation (20). The detergent action of shampoos solubilizes the viral lipid bilayer, effectively eliminating or reducing viral infectivity regardless of the shampoo or co-wash formulation used.

The second part of the study aimed to assess the fomite transmission risk associated with virus- exposed hair and the efficacy of mechanical removal of non-enveloped viruses (BRBV) from different hairstyles and hair types by showering, using pre-tested (liquid suspension assay) hair products. We systematically evaluated different styles of synthetic and human hair under controlled viral exposure and washing conditions. Goat tongue epithelial cells were identified, for the first time, as excellent targets for BRBV, as they exhibit clear cytopathic effects even at the early stage of infection and a low viral dose. Following characterization of the infection model, we developed a novel BSL-2 experimental set up to be conducted within the biosafety cabinet to safely study the effectiveness of shower-out practices in the mechanical removal of non-enveloped virus from hair. Some challenges were identified, including the choice of hair types, due to the intrinsic cytotoxicity in downstream cell culture and the need for two operators to perform the experiments. An additional challenge inherent to hair as a sample type was the development of a reliable method to recover viral particles efficiently following washing. We also identified a formulation-related limitation associated with the Design Essentials Natural Almond and Avocado shampoo brand, which produced mild cytotoxicity in most experiments, independent of hair type. This effect was most likely due to incomplete rinsing and/or high oil percentage in the hair product, resulting in residual product that interfered with cell culture assays. However, BRBV-specific CPE were distinguishable from cytotoxicity, and BRBV infection occurred even in mildly altered cells, Fig. 2B.

Notably, viral exposure of the mannequin head was highly realistic, as evidenced by the fact that in three independent experiments; some hair strands of the viral exposure control were negative. The hair strands that did not show infection at the end of passage 2 come from the hair strand collected from the back; this likely reflects the static positioning of wigs during exposure, which was intended to mimic natural scenarios rather than ensure uniform viral inoculation, while at the same time providing sufficient viral exposure to produce a positive result in at least two of the three strands of hair collected from the viral exposure control. In addition, each type of hair was exposed to a slightly different dose of BRBV resulting in a variable amount of viral load in the exposure control (harvested at day 0, Table S3) and the positive results (Tables S4 and S5) of the showered hairs. Importantly, no correlation could be found between the initial amount of viruses used to expose the hair, nor the titer of the virus recovered from the hair used as an exposure control and the final assessment of hair type positivity.

It is important to mention that we could not pick a specific dose of BRBV that would have mocked what is found in FMDV-infected animals; in fact to our knowledge, this information is lacking. Overall, the shower-out practice, which consisted of 3 min of shampoo massaging followed by 2 min of water rinsing, regardless of hairstyle or type, resulted in full decontamination by mechanical rinsing with shampoo and, surprisingly, even with water alone, with some exceptions. We found that one of the three strands (the front) of the synthetic curly/kinky hair tested positive only following co-wash treatment. This positive result is likely multifactorial. One contributing factor may be the wig itself, which features a high volume of hair. Another factor could be the hair type: curly/kinky hair forms a dense, interwoven network that can hinder thorough rinsing, leaving certain areas less accessible during washing. Finally, the hair product itself, the co-wash, contained very low levels of surfactant ingredients and was instead enriched with oils. These oils may have trapped viral particles during the exposure period, thereby reducing the efficiency of subsequent mechanical removal. It is notable, however, that no other hairstyles and types produced positive results with this same co-wash product.

Another positive result was observed with the mannequin head equipped with 100 synthetic braids, which were washed either with TRESemmé shampoo or with water alone. When compared to the mannequin head fitted with the same type of braids but in fewer amounts (around 45), no positive results were obtained. This suggests that the total volume of hair, rather than the hairstyle itself, is a critical determinant of residual viral contamination. The higher number of braids increases the time and effort required for thorough washing, and inadequate shampoo or water penetration may leave areas untreated. It is worth mentioning that only one strand of hair out of three and one type of shampoo resulted in a positive result. Indeed, although TRESemmé was the only shampoo to show mild *in vitro* inactivation of BRBV, the persistence of virus in samples from the 100-braid mannequin head likely reflects limited shampoo access to all hair regions. This interpretation is further supported by the water-only controls: two of the three samples from the 100 braids tested positive, while the 45 braids mannequin washed under identical conditions did not yield any positivity. Interestingly, the human hairs never showed CPEs. We cannot confidently infer that human hair is easier to wash than synthetic hair because the hairstyles differed slightly. For example, the human straight hair after the washing pretreatment becomes slightly wavy; the water might have disrupted the hydrogen bonds revealing the hair’s natural wavy pattern, whereas the synthetic counterpart maintains its straightness. Another example is the synthetic curly/kinky hair that was twice as voluminous and curlier than the human counterpart. Similarly, the synthetic loose loc wigs had more locs (150) than the mohawk-style human locs, which had 35 strands of hair.

Overall, the use of synthetic and human hair models provided a valuable system for evaluating the relative fomite risk associated with different hairstyles (straight, curly/kinky, locs, and braids) and, in general, across different hair types (synthetic and human). Although we included four types of artificial hair and three types of human hair, washed with three kinds of shampoos and one type of co-wash, we acknowledge that the variability of human hair and synthetic extensions among real-world laboratory personnel is highly diverse, and our findings represent only a limited perspective.

Taken together, our findings suggest that shampoo choice is not the primary determinant of viral removal in high-containment personnel shower-out practices. In fact, even a 2-min water rinse alone was sufficient to mechanically remove viral particles from most of the mannequin heads tested. Instead, the critical factor appears to be the volume and density of the hair: synthetic curly/kinky extensions, which were denser than human curly/kinky hair, were more likely to yield positive results, as were the 100 synthetic braids compared to 45. Moreover, due to the positive result in the co-wash treatment, attention must be put on the presence of oil-based product in the hair of personnels before entering the high containment facilities that could potentially interfere with virus mechanical removal during showering. We cannot completely rule out that a higher initial viral load per hair strand may affect BRBV mechanical removal during showering. Notably, the 100-braids hairstyle tested positive after both a single shampoo treatment and a water rinse alone. This hairstyle also showed the highest viral load among the hair samples in the no-rinse exposure control.

These data highlight the importance of thorough washing techniques, particularly when large hair volumes are present. Special attention should, therefore, be directed to decontaminating dense hair to minimize transmission risk in high-containment settings.

## MATERIALS AND METHODS

### Propagation of goat tongue and SW-13 cells

Goat tongue epithelial (ZZ-R-127) cells (Friedrich Loeffler Institute, Germany) and human adrenal gland (SW-13) cells (adapted at CDC, Atlanta, GA) were used at passage 51–57 and passage 7–15, respectively. Goat tongue cells were propagated in growth medium composed of a 1:1 ratio of EMEM with Earle’s salts + L-glutamine (Corning) and high-glucose DMEM + L-glutamine (Gibco), supplemented with 5%–7% inactivated fetal bovine serum (ATCC), 1% Antibiotic-Antimycotic 100× (Gibco), 1% of 100 mM sodium pyruvate (Sigma), and 1% non-essential amino acids 100 mM (Sigma). Goat tongue cells were maintained at 37°C with 5% CO₂ in T150 flasks. SW-13 cells were propagated in high-glucose DMEM + L-glutamine (Gibco), 5% inactivated fetal bovine serum (Gibco), and 1× penicillin/streptomycin 100× (Gibco). SW-13 cells were maintained at 37 °C with 5% CO₂ in T75 flasks.

### Propagation of Bovine Rhinitis B virus and Hazara virus

BRBV strain EC-11 (ATCC VR-1806) was obtained from the American Type Culture Collection (Manassas, VA), and HAZV strain JC280 was obtained from the World Reference Center for Emerging Viruses and Arboviruses collection from the University of Texas Medical Branch.

To propagate BRBV and HAZV, the archived 1:10 viral stock was diluted to achieve MOIs of 0.2 or 0.01 for each virus. Goat tongue ZZ-R and SW-13 cells grown in T150 flasks were rinsed with 1× PBS and inoculated with 5 mL suspension of BRBV or HAZV in EMEM or DMEM, respectively. Flasks were rocked every 15–20 min for 1 h, at 33°C and 5% CO₂ for BRBV and at 37°C and 5% CO₂ for HAZV. After adsorption, 25 mL of the respective media were added, and the cell monolayers were incubated for an additional 24 h and checked daily for cytopathic effect (CPE). In addition, BRBV-RNA was monitored daily from cell culture supernatants by real-time PCR detection of the 3D Pol gene on the Light Cycler 96 using primers and probe previously developed by Kishimoto and others (25). Cells were harvested and frozen at –80°C when 70%–80% CPE was observed. After thawing, cell suspensions were centrifuged at 4°C for 5 min at 2,000 rpm; to the supernatant, inactivated FBS at a final concentration of 10% and 0.01 M Tris were added, the final suspension was aliquoted into individual cryovials and stored at –80 °C until needed.

### Titration of Bovine Rhinitis B and Hazara virus

Virus stock or samples were titrated using the 50% tissue culture infectious dose (TCID50) method. ZZ-R Goat cells or SW-13 were seeded at a density of 2 × 10^5^ cells per well in 100 μL growth medium in 96-well plates and allowed to reach 90%–100% confluence over 4–5 days (BRBV) or 2–3 days (HAZV). Once confluent, the growth medium was removed and replaced with fresh medium. Serial 10-fold dilutions from 10^−1^ to 10^−7^ or 10^−8^ were prepared in the appropriate medium (EMEM5-ZZ-R or DMEM5-SW-13) in a final volume of 300 µL, and 50 or 100 µL of each sample dilution was inoculated per well for a total of 4–6 replicate wells. Cultures were incubated for 7 days at 33°C (ZZ-R) or 37°C (SW-13) with 5% CO_2_ in a humidified incubator. After 7 days, plates were fixed and stained with 100 µL of 1× crystal violet in 10% neutral buffered formalin for 1 h at room temperature. The fixative/stain was discarded, and plates were gently rinsed with water to remove excess stain and then dried in a biosafety cabinet overnight. Plates were viewed under white light, and the presence or absence of CPE was recorded, and titers were calculated via TCID50/mL using the Reed and Muench method (33).

### Liquid suspension assay of Bovine Rhinitis B and Hazara viruses and hair products

To test the inactivation ability of different hair products, goat tongue ZZ-R or SW13 cells were seeded as described above. Briefly, cells were seeded into 96-well plates with 100 μL of medium and allowed to reach 90%–100% confluence over 4–5 days (BRBV) 2–3 days (HAZV). Four shampoos: TRESemmé(1), Ginger Lily Farms Morocco Argan Oil (2), Design Essentials Natural Almond and Avocado (3) and Gojo SPA Bath and Body SPA Bath (4), one co-wash, Design Essentials Natural Almond and Avocado (5), a hair product considered a trade-off between a detergent and conditioners and three leave-in conditioners, Design Essentials Natural Almond and Avocado (6), Melanin multi-use softening (7), Hair rules Nourishment (8) were tested. The hair product selection was based on BSL-4 lab users’ preference, facility recommendations, and/or products used at Plum Island Animal Disease Center (PIADC). Ten milliliters of fresh hair product suspension was prepared for each biological replicate as follows: shampoos, co-wash, and conditioners were transferred using a 5 mL Pasteur pipette with the tip cut, weighed, and diluted in deionized water at a ratio of 1:10. A volume of 300 μL of this suspension was then transferred to a 1.5 mL tube. For the liquid suspension inactivation assay, 300 μL of the hair product suspension and 300 μL of viral solution (5.3 log TCID50/mL for BRBV and 4.5 log TCID50/mL for HAZV) were mixed and incubated for 3 min. After incubation, 30 μL of the hair product-virus suspension was diluted in 270 μL of media (EMEM, 5% FBS, 1% anti/anti) and serially diluted until 10^−6^. Before inoculation, the media in the two cells were replaced with fresh medium (100 μL), and four to six wells per hair product were inoculated with 50 μL of the undiluted or serially diluted mixture, without detergent removal. Since hair products are cytotoxic, cytotoxicity plates for each hair product were prepared in parallel using media instead of viral solution. A positive control (media and virus) and an inactivation control (5% bleach and virus) were also included. Plates were incubated at 33°C and 37°C for BRBV and HAZV, respectively, for 7 days and then fixed with 100 μL of 1% crystal violet and 10% formalin for 1 h. Plates were washed four times in water and allowed to dry overnight (O/N). Two independent biological replicates were performed for each virus type. Viral titers (TCID50) were calculated using the Reed and Muench method, and the reduction of titer due to hair product treatment relative to the positive control was calculated using this formula: (Positive Control Log_10_ TCID50 – Hair Product Log_10_ TCID50)/Positive Control Log_10_ TCID50. The list of hair products, their chemical composition, and the specific weights used for the experiments is provided in Table S1.

### Minimum detectable amount of BRBV recovery from synthetic hair

To measure the sensitivity of the BRBV and tongue goat cells model, a sensitivity test aimed at identifying the minimum detectable amount of BRBV able to infect ZZ-R goat tongue cells, cells were seeded into T25 flasks and allowed to reach 80% confluence over 5–7 days. On the day of infection, a 5.3 log TCID50 virus solution was serially diluted in media (EMEM, 5% FBS, 1% anti/anti) to 10^−5^ in a final volume of 1 mL. Two hair loops of synthetic curly/kinky hair (each 6 inches long) were placed in a plastic reservoir and exposed to 1 mL of each dilution in duplicates. The exposed hairs were left to dry for 30 min, the hair loops were squeezed in a plastic bag, containing 5 mL of media and with the resultant inoculum cells were infected for 1 h, then the inoculum was removed, and 5 mL of fresh EMEM5 was added. Negative and positive controls were included: 3 mL EMEM5 plus 2 mL sink water (harmful) and 30 µL virus plus 5 mL EMEM5 (positive). Cells were checked for CPE for 1 week. After 7 days, 50% of the supernatant was passed into 80%–90% fresh confluent cells, and 1.5 mL was saved as backup material. After another 7 days, 1 mL of the passage-2 supernatant was saved for back-titration and 1 mL as a backup. The experiment was done twice using curly/kinky synthetic hair.

### Mechanical removal assay of non-enveloped virus from unbound and bound synthetic and human hairs by showering

To determine the risk of post-shower transmission of viruses across different hairstyles and hair types, goat tongue ZZ-R cells were seeded into T25 flasks with 5 mL medium and allowed to reach 80%–90% confluence over 5–7 days. Multiple hairstyles and types were used. The hair styles of this study were determined by the Andre Walker Hair Typing System, also known as the Hair Chart (34). The Andre Walker Hair system is based on 12 types of hair—whether they are 1 (Straight), 2 (Wavy), 3 (Curly), or 4 (Coiled/Kinky), and whether the strands are A (fine), B (medium, some volume), or C (coarse, thick, tight). Synthetic and human hairs, as mannequin, wigs, or extensions, were purchased from individual vendors through Amazon.com. Two types of hair were used, synthetic and human hair. For the synthetic type of hairs, four different hairstyles were chosen, straight (1A), curly/kinky (4A), loose locs (4A), and two amounts of the same type of braids, 45 and 100. For the human type of hairs, three styles were picked, straight/wavy (2A), curly/kinky (3B), and locs (4B). All the hair were kept 12 inches long. Hair mannequin, wigs, or extensions were pre-washed by hand or in an industrial dishwasher with only water and then air-dried overnight. Once dry, the hair was sewn or stapled onto a bald mannequin head (details in Table S2). Four different types of hair products previously tested in the liquid suspension assay were used: three shampoos TRESemmé (1), Ginger Lily Farms Morocco Argan Oil (2), Design Essentials Natural Almond (3) and one co-wash, Design Essentials Natural Almond and Avocado (4). The hair products were diluted in a final volume of 5 mL in a 1:5 shampoo suspension, prepared by weighing the shampoo and adding 5 mL deionized water in a white reservoir.

On the day of the experiment, six mannequin heads were exposed to a range of 5.5 × 10^4^ to 3.1 × 10^6^ TCID50 using an intranasal mucosal atomization device (Teleflex) attached to a 3 mL syringe to aerosolize the suspension evenly. Depending on the experiment, from 500 to 800 µL of virus per head was applied to ensure complete coverage, details on the titer of BRBV used per experiment are in Table 1, the specific volume of the viral treatment per hair type, and the titer of each strand of exposure control—no rinse is listed in Table S3. Each head was placed inside a biosafety cabinet, sprayed over a white autoclave bin, and then moved to a second bin. After exposure, heads were left to dry for 30 min to 1 h, and the BRBV virus used to expose the head was also left at room temperature for the same time as the heads. One by one, heads were locked on a hook at the short side of the bin, pre-labeled with a liquid-measurement tape, and a plastic sheet was placed to prevent splashing. Before shampoo/inactivation treatment, each head was rinsed under running water for 30 s at a flow rate of 4–5 L/min (28). The hair product suspension was applied in multiple spots and massaged for 3 min and then rinsed for 2 min. A control omitted shampoo called “water rinse” was done, and heads were rinsed for 2 min and 30 s. A further control for viral exposure included a head exposed to the virus and immediately harvested for hair samples, without a water rinse. A cytotoxic control used uninfected hair wet-harvested to verify that cell death was virus-related. Each hairstyle was treated using the same washing and rinsing technique, with only a few specific exceptions. The general massaging and rinsing technique were as follows: massaging with both hands, working thoroughly with the fingers on the scalp, ensuring uniform time and pressure. The rinsing was less active; the water was allowed to run out of the hair by actively squeezing the hair with both hands from the scalp to the ends. For the voluminous synthetic curly/kinky hair, twice the amount of hair products was used to ensure total coverage; however, it seems that more massaging and rinsing were needed. Loose locs, locs, and braids needed to be massaged on the scalp, as well as the single strand of hair. Rinsing required single strand squeezing to ensure proper washing of the hair. After rinsing, hair strands were cut from the front, back, and center of each head and placed into plastic bags. Human Locs and synthetic 100 braids were rinsed for 2 min and a half instead of 2 min. Hair samples were kept refrigerated until all hair strands from other mannequin heads were collected. Each hair sample in the bag was reconstituted with 7 mL EMEM5 and agitated at 1,000 rpm for 5 min in a vortex mixer to facilitate elution of virus from the hair. Subsequently, bags containing hair strands were gently squeezed by hand to transfer the liquid into a 50 mL tube and then filtered through a 0.2 µm filter to prevent fungal contamination. To quantify the BRBV amount, 500 μL of harvested material from each hair treatment and from the virus used to spray the head was saved. The remaining 5–7 mL was inoculated into T25 flasks for infection, which was performed as follows: 1 h absorption, then the inoculum was removed, and fresh 5 mL of EMEM5 was added. Negative and positive controls were included: 3 mL EMEM5 plus 2 mL sink water (negative) and 30 µL virus plus 5 mL EMEM5 (positive). For the human hair wavy and kinky, the inoculum was divided in the T25, and 3.5 mL of media was added. Cells were checked for CPE for 1 week. After 7 days, 50% of the supernatant was passed into 85%–90% fresh confluent cells, and 0.5 mL was saved as backup material. After another 7 days, 0.5 mL of the passage 2 supernatant was saved for back-titration and 0.5 mL as a backup.

## Supplementary Material

Reviewer comments

## Data Availability

Data of this study are available within the article and its supplemental material.

## SUPPLEMENTAL MATERIAL

Figure S1 and Tables S1 to S4. spectrum.00387-26-s0001.docx Figure S1: Hair products and hairstyles. Table S1: Hair Product Characteristics. Table S2: Details of brands of hair and pre-washed conditions and washing techniques. Table S3: Results of the end of passage 1 of mechanical removal of non-enveloped virus from synthetic and human hairs by showering. Table S4: Results of the end of passage 2 of mechanical removal of non-enveloped virus from synthetic and human hairs by showering.

OPEN PEER REVIEW reviewer-comments.pdf An accounting of the reviewer comments and feedback.

ASM does not own the copyrights to Supplemental Material that may be linked to, or accessed through, an article. The authors have granted ASM a non-exclusive, world-wide license to publish the Supplemental Material files. Please contact the corresponding author directly for reuse.
