# Supplementary material for: Evidence-based recommendations for shower-out practices: assessing virus removal from hair using a surrogate non-enveloped virus
Source: Microbiol Spectr. 2026 Jul 13;14(8):e00387-26. doi: 10.1128/spectrum.00387-26 (PMC13436421; doi:10.1128/spectrum.00387-26)
Supplement: Supplementary file 1 [file reviewer-comments.pdf]

AMERICAN SOCIETY FOR MICROBIOLOGY | Microbiology Spectrum

Evidence-based recommendations for shower-out practices: assessing virus removal from hair using a surrogate non-enveloped virus

Anastasia Accoti, Maria Cajimat, Catherine Olal, Krystle Hensley, Joseph Kozlovac, and Dennis Bente

Corresponding Author(s): Anastasia Accoti, The University of Texas Medical Branch at Galveston Store

**Review Timeline:**		
	Submission Date:	February 9, 2026
	Editorial Decision:	March 6, 2026
	Revision Received:	May 13, 2026
	Accepted:	May 30, 2026

Editor: Kessendri Reddy

Reviewer(s): Disclosure of reviewer identity is with reference to reviewer comments included in decision letter(s). The following individuals involved in review of your submission have agreed to reveal their identity: Aldo Dekker (Reviewer #1); Rebecca Moritz Moritz (Reviewer #2)

Transaction Report:

(Note: With the exception of the correction of typographical or spelling errors that could be a source of ambiguity, letters and reports are not edited. The original formatting of letters and referee reports may not be reflected in this compilation.)

DOI: https://doi.org/10.1128/spectrum.00387-26

Re: Spectrum00387-26 (**Evidence-based recommendations for shower-out practices: assessing virus removal from hair using a surrogate non-enveloped virus**)

Dear Dr. Anastasia Accoti:

Thank you for the privilege of reviewing your work. Below you will find my comments, instructions from the Spectrum editorial office, and the reviewer comments.

• References: Please double check these and place into the appropriate format.
• Introduction and throughout: Biosafety level is sometimes abbreviated as BLS (line 50) and sometimes as BSL (line 60). Please harmonize throughout the manuscript.
• Introduction, Line 88: please write as "1960s", as "60s" sounds quite colloquial.
• Introduction, Lines 111-119: Please remove this and place it into Materials and Methods, and rewrite in past tense.
• Introduction, Lines 120-121: Please delete, it is redundant.
• Results: As also mentioned by Reviewer 1, method details are included in the results. Please separate this into the relevant sections.

Please return the manuscript within 60 days; if you cannot complete the modification within this time period, please contact me. If you do not wish to modify the manuscript and prefer to submit it to another journal, notify me immediately so that the manuscript may be formally withdrawn from consideration by Spectrum.

Revision Guidelines
To submit your modified manuscript, log into the submission site at https://spectrum.msubmit.net/cgi-bin/main.plex. Go to Author Tasks and click the appropriate manuscript title to begin. The information you entered when you first submitted the paper will be displayed; update this as necessary. Note the following requirements:

• Upload point-by-point responses to the issues raised by the reviewers in a file named "Response to Reviewers," NOT in your cover letter.
• Upload a compare copy of the manuscript (without figures) as a "Marked-Up Manuscript" file.
• Upload a clean .DOC/.DOCX version of the revised manuscript and remove the previous version.
• Each figure must be uploaded as a separate, editable, high-resolution file (TIFF or EPS preferred), and any multipanel figures must be assembled into one file.
• Any supplemental material intended for posting by ASM should be uploaded with their legends separate from the main manuscript. You can combine all supplemental material into one file (preferred) or split it into a maximum of 10 files with all associated legends included.

For complete guidelines on revision requirements, see our Submission and Review Process webpage. Submission of a paper that does not conform to guidelines may delay acceptance of your manuscript.

Data availability: ASM policy requires that data be available to the public upon online posting of the article, so please verify all links to sequence records, if present, and make sure that each number retrieves the full record of the data. If a new accession number is not linked or a link is broken, provide Spectrum production staff with the correct URL for the record. If the accession numbers for new data are not publicly accessible before the expected online posting of the article, publication may be delayed; please contact production staff (Spectrum@asmusa.org) immediately with the expected release date.

Publication Fees: For information on publication fees and which article types are subject to charges, visit our website. If your manuscript is accepted for publication and any fees apply, you will be contacted separately about payment during the production process; please follow the instructions in that e-mail. Arrangements for payment must be made before your article is published.

ASM Membership: Corresponding authors may join or renew ASM membership to obtain discounts on publication fees. Need to upgrade your membership level? Please contact Customer Service at Service@asmusa.org.

The ASM Journals program strives for constant improvement in our submission and publication process. Please tell us how we can improve your experience by taking this quick Author Survey.

Thank you for submitting your paper to Spectrum.

Sincerely,
Kessendri Reddy
Editor
Microbiology Spectrum

Reviewer #1 (Comments for the Author):

Evidence-based recommendations for shower-out practices: assessing virus removal from hair using a surrogate non-enveloped virus

Anastasia Accoti, Maria N.B. Cajimat, Catherine Olal, Krystle Hensley, Joseph Kozlovac, and Dennis A. Bente

This is an extremely relevant piece of work. I'm impressed with all the work, especially using different types of hair is interesting.

However, the presentation of material and methods, results and discussion needs improvement. Do not repeat material and methods in the results section, the results section can be much shorter. Do not repeat introduction and results in the discussion section. In the discussion section it would be interesting to discuss pH sensitivity of bovine rhinitis B virus. Foot-and-mouth disease virus is pH sensitive; it falls apart below neutral pH and is almost fully inactivated at pH 5. (Bachrach, H. L., Breese, S. S., Callis, J. J., Hess, W. R., Patty, R. E. 1957. Inactivation of Foot-and-Mouth disease virus by pH and Temperature changes and by Formaldehyde. Proc. Soc. Exp. Biol. and Med. 95; 147-152). I could not find such a paper on bovine rhinovirus B, I assumed acid sensitivity is similar but based on the pH of the products it seems that virus can survive better at low pH. Interestingly the pH of TREsemmé is the lowest (suppentary table 1) and inactivates better (figure 1).

The materials and methods section should be written with more clarity and the Q-RT-PCR that is mentioned in the results should also be mentioned in the M&M section.

I would assume that from each wig hairs were collected before washing to estimate the virus concentration. I could not find this in the materials and methods section nor in the results section, but I see results in the supplementary tables. I do not see virus titres though, only detection of virus, then it would be good to discuss the virus titres found after washing in relation to the virus titre applied (which should be mentioned). The application of 0.5 to 0.8 ml to a whole wig is relatively a small amount.

Please indicate in the discussion that only non-enveloped virus was tested for survival after showering, as the enveloped viruses were already inactivated by Shampoo.

Specific comments:

The ZZ-R (127) cells are described in https://www.cellosaurus.org/CVCL_5418
Abstract:
Line 25 and 281: delete "highly". Contagiousness is not solely a characteristic of the virus, but also of the host density. In endemic settings the R-naught estimates are less than 1.5, which is not highly contagious.

Introduction:
Line 67: delete "one of the most" (see above not true and it is not a competition). You could stress the economic consequences more.

Line 73 - 75: Please consider deleting this sentence. The support of airborne transmission is mainly based on believes and not on actual data. In fact, de Bouma et al. 2004 showed that calves housed in the same stable did not transmit when kept in in boxes, whereas Orsel et al. 2004 showed that within the same age group transmission occurred when animals were freely roaming. This shows that the contribution of airborne transmission in cattle is limited. The work of Sorensen et al. 2000 showed that previous results with Gausian spread models could only be reproduced when more than 100 times more virus output was used (the strain in Isle of Wight did only produce 6.2 log virus per day and not 8.6), extreme susceptibility of cattle was entered in the model and stable conditions above seawater were exaggerated. Although the author did not conclude that previous estimates were wrong, the data clearly show that previous estimates were wrong.
Bouma, A., Dekker, A., de Jong, M. C. M. 2004. No foot-and-mouth disease virus transmission between individually housed calves. Vet. Microbiol. 98(1); 29-36.
Orsel, K., Dekker, A., Bouma, A., Stegeman, J. A., de Jong, M. C. M. 2005. Vaccination against foot and mouth disease reduces virus transmission in groups of calves. Vaccine 23(41); 4887-4894.
Sorensen, J. H., Mackay, D. K. J., Jensen, C. O., Donaldson, A. I. 2000. An integrated model to predict the atmospheric spread of foot-and-mouth disease virus. Epidemiology-and-Infection 124(3); 577-590.

Line 111-119: End the introduction with a clear objective, e.g. the objective of the current study was the evaluation of virus inactivation by different hair products (shampoo and conditioner) as well as the effect of washing hair on virus survival. The product names will be mentioned in material and methods. Be concise be precise.

Line 120-121: Delete these lines, they can be used in the discussion.

Line 165: Suggestion for section title: Evaluation of titration of bovine rhinitis B Virus in goat tongue cells

Line 166-167: can be deleted

Line 170: specify the species from which the kidney cells were cultured (primary porcine kidney cell, primary bovine kidney cells or primary ovine kidney cells).

Line 173: after "tongue cells" add (ZZ-R from Friedrich Loeffler Institute, Germany)

Results:
The whole result section contains to much material and methods and is not easy to read. Please rewrite.

Line 200 to 210: check the use of the word "for". Check the English of this part.

Line 211 to 214: It is not clear how "cosmetology mannequin head" is different from a bald head with a wig.

Line 227: How many hairs are in a strand?

Line 227: replace "large" with "long", at least I think that is what you mean.

Line 227: I do not understand the reference to number 2 (Biosafety in Microbiological and Biomedical Laboratories (BMBL) 6th Edition)

Line 228: the hair is not filtered through 200 nm filter, but the eluted material.

Discussion:
The discussion should not repeat "Introduction", "Materials and Methods" and "Results". Start the discussion with the objective (often the last sentence in the introduction) and then discuss the results.

Line 288: Replace "direct-contact liquid suspension assay" by "quantitative suspension test", this is the term also used in norm for disinfectant testing, e.g. EN 14675:2015 - Quantitative suspension test for the evaluation of virucidal activity of chemical disinfectants and antiseptics used in the veterinary area (https://microbe-investigations.com/disinfectant-testing/disinfectant-antiviral/en-14675-virucidal-testing-of-veterinary-disinfectants/)

Materials and methods:
I see results of Q-RT-PCR but no reference to the method used in materials and methods. Please add a reference.
Line 443 and 476: Reed and Muench is not a good method. It assumes results that were not observed. Spearman Karber is superior "The time has come for a change: under the most favorable conditions, neither [the Reed-Muench nor the Dragstedt-Behrens] method is as precise as the Spearman-Karber, and often Spearman-Karber is markedly superior.... Except as part of statistical history, both methods should be forgotten."
Myers, L. E., McQuay, L. J., Hollinger, F. B. 1994. Dilution assay statistics. J. Clin. Microbiol. 32(3); 732-739.

Line 541: It is not clear how many hairs were collected. In the results section you state 2 inch (= 5 cm in SI units) of hair was collected. Not only the length, but also the number of hairs might influence the result.

Lin 542 - 543: With "2 minutes and a half" do you mean 2.5 minutes? Which would be clearer to the reader.

Line 554: did you check daily or only once during that week?

Figures:
The colour of Design Essential conditioner in figure 1 is different in the graph compared to the legend.

Supplementary tables and figures:
The difference between supplementary table 3 and 4 is very small. It is strange that "Braids 100" has a positive result with TREsemmé-Shampoo which is not found in the second passage. Could this be toxic effect on the cells and no virus? TREsemmé inactivated the virus rather well in the suspension test, so it would be logical that it works best in the washing test.

References:
The reference list needs a lot of attention. E.g. number 6 should read
Bøtner, A., Belsham, G. J. 2012. Virus survival in slurry: Analysis of the stability of foot-and-mouth disease, classical swine fever, bovine viral diarrhoea and swine influenza viruses. Vet. Microbiol. 157(1-2); 41-49.

Reviewer #2 (Comments for the Author):

Well done paper- the experiments and methodologies were well thought out

Evidence-based recommendations for shower-out practices: assessing virus removal from hair using a surrogate non-enveloped virus

Anastasia Accoti, Maria N.B. Cajimat, Catherine Olal, Krystle Hensley, Joseph Kozlovac, and Dennis A. Bente

This is an extremely relevant piece of work. I'm impressed with all the work, especially using different types of hair is interesting.

However, the presentation of material and methods, results and discussion needs improvement. Do not repeat material and methods in the results section, the results section can be much shorter. Do not repeat introduction and results in the discussion section. In the discussion section it would be interesting to discuss pH sensitivity of bovine rhinitis B virus. Foot-and-mouth disease virus is pH sensitive; it falls apart below neutral pH and is almost fully inactivated at pH 5. (Bachrach, H. L., Breese, S. S., Callis, J. J., Hess, W. R., Patty, R. E. 1957. Inactivation of Foot-and-Mouth disease virus by pH and Temperature changes and by Formaldehyde. Proc. Soc. Exp. Biol. and Med. 95; 147-152). I could not find such a paper on bovine rhinovirus B, I assumed acid sensitivity is similar but based on the pH of the products it seems that virus can survive better at low pH. Interestingly the pH of TREsemmé is the lowest (suppentary table 1) and inactivates better (figure 1).

The materials and methods section should be written with more clarity and the Q-RT-PCR that is mentioned in the results should also be mentioned in the M&M section.

I would assume that from each wig hairs were collected before washing to estimate the virus concentration. I could not find this in the materials and methods section nor in the results section, but I see results in the supplementary tables. I do not see virus titres though, only detection of virus, then it would be good to discuss the virus titres found after washing in relation to the virus titre applied (which should be mentioned). The application of 0.5 to 0.8 ml to a whole wig is relatively a small amount.

Please indicate in the discussion that only non-enveloped virus was tested for survival after showering, as the enveloped viruses were already inactivated by Shampoo.

Specific comments:

The ZZ-R (127) cells are described in https://www.cellosaurus.org/CVCL_5418
Abstract:
Line 25 and 281: delete "highly". Contagiousness is not solely a characteristic of the virus, but also of the host density. In endemic settings the R-naught estimates are less than 1.5, which is not highly contagious.

Introduction:
Line 67: delete "one of the most" (see above not true and it is not a competition). You could stress the economic consequences more.

Hair Treatment	Synthetic Hair					Human Hair		
	Straight	Kinky	Loose Locs	45 Braids	100 Braids	Straight/ Wavy	Kinky	Locs
Ginger-Shampoo	0/3	0/3	0/3	0/3	0/3	0/3	0/3	0/3
TREsemmé-Shampoo	0/3	0/3	0/3	0/3	1/3*	0/3	0/3	0/3
De Essentials-Shampoo	0/3	0/3	0/3	0/3	0/3	0/3	0/3	0/3
De Essentials-Co-wash	0/3	1/3*	0/3	0/3	0/3	0/3	0/3	0/3
Water	0/3	0/3	0/3	0/3	2/3#	0/3	0/3	0/3

Line 73 – 75: Please consider deleting this sentence. The support of airborne transmission is mainly based on believes and not on actual data. In fact, de Bouma et al. 2004 showed that calves housed in the same stable did not transmit when kept in in boxes, whereas Orsel et al. 2004 showed that within the same age group transmission occurred when animals were freely roaming. This shows that the contribution of airborne transmission in cattle is limited. The work of Sorensen et al. 2000 showed that previous results with Gausian spread models could only be reproduced when more than 100 times more virus output was used (the strain in Isle of Wight did only produce 6.2 log virus per day and not 8.6), extreme susceptibility of cattle was entered in the model and stable conditions above seawater were exaggerated. Although the author did not conclude that previous estimates were wrong, the data clearly show that previous estimates were wrong.

Bouma, A., Dekker, A., de Jong, M. C. M. 2004. No foot-and-mouth disease virus transmission between individually housed calves. Vet. Microbiol. 98(1); 29-36.

Orsel, K., Dekker, A., Bouma, A., Stegeman, J. A., de Jong, M. C. M. 2005. Vaccination against foot and mouth disease reduces virus transmission in groups of calves. Vaccine 23(41); 4887-4894.

Sorensen, J. H., Mackay, D. K. J., Jensen, C. O., Donaldson, A. I. 2000. An integrated model to predict the atmospheric spread of foot-and-mouth disease virus. Epidemiology-and-Infection 124(3); 577-590.

Line 111-119: End the introduction with a clear objective, e.g. the objective of the current study was the evaluation of virus inactivation by different hair products (shampoo and conditioner) as well as the effect of washing hair on virus survival. The product names will be mentioned in material and methods. Be concise be precise.

Line 120-121: Delete these lines, they can be used in the discussion.

Line 165: Suggestion for section title: Evaluation of titration of bovine rhinitis B Virus in goat tongue cells

Line 166-167: can be deleted

Line 170: specify the species from which the kidney cells were cultured (primary porcine kidney cell, primary bovine kidney cells or primary ovine kidney cells).

Line 173: after "tongue cells" add (ZZ-R from Friedrich Loeffler Institute, Germany)

Results:

The whole result section contains to much material and methods and is not easy to read. Please rewrite.

Line 200 to 210: check the use of the word "for". Check the English of this part.

Line 211 to 214: It is not clear how "cosmetology mannequin head" is different from a bald head with a wig.

Line 227: How many hairs are in a strand?

Line 227: replace "large" with "long", at least I think that is what you mean.

Line 227: I do not understand the reference to number 2 (Biosafety in Microbiological and Biomedical Laboratories (BMBL) 6th Edition)

Line 228: the hair is not filtered through 200 nm filter, but the eluted material.

Discussion:
The discussion should not repeat "Introduction", "Materials and Methods" and "Results". Start the discussion with the objective (often the last sentence in the introduction) and then discuss the results.

Line 288: Replace "direct-contact liquid suspension assay" by "quantitative suspension test", this is the term also used in norm for disinfectant testing, e.g. EN 14675:2015 - Quantitative suspension test for the evaluation of virucidal activity of chemical disinfectants and antiseptics used in the veterinary area (https://microbe-investigations.com/disinfectant-testing/disinfectant-antiviral/en-14675-virucidal-testing-of-veterinary-disinfectants/)

Materials and methods:
I see results of Q-RT-PCR but no reference to the method used in materials and methods. Please add a reference.
Line 443 and 476: Reed and Muench is not a good method. It assumes results that were not observed. Spearman Karber is superior "The time has come for a change: under the most favorable conditions, neither [the Reed-Muench nor the Dragstedt-Behrens] method is as precise as the Spearman-Karber, and often Spearman-Karber is markedly superior.... Except as part of statistical history, both methods should be forgotten."
Myers, L. E., McQuay, L. J., Hollinger, F. B. 1994. Dilution assay statistics. J. Clin. Microbiol. 32(3); 732-739.

Line 541: It is not clear how many hairs were collected. In the results section you state 2 inch (= 5 cm in SI units) of hair was collected. Not only the length, but also the number of hairs might influence the result.

Lin 542 – 543: With "2 minutes and a half" do you mean 2.5 minutes? Which would be clearer to the reader.

Line 554: did you check daily or only once during that week?

Figures:
The colour of Design Essential conditioner in figure 1 is different in the graph compared to the legend.

Supplementary tables and figures:

The difference between supplementary table 3 and 4 is very small. It is strange that "Braids 100" has a positive result with TREsemmé-Shampoo which is not found in the second passage. Could this be toxic effect on the cells and no virus? TREsemmé inactivated the virus rather well in the suspension test, so it would be logical that it works best in the washing test.

References:

The reference list needs a lot of attention. E.g. number 6 should read

Bøtner, A., Belsham, G. J. 2012. Virus survival in slurry: Analysis of the stability of foot-and-mouth disease, classical swine fever, bovine viral diarrhoea and swine influenza viruses. Vet. Microbiol. 157(1–2); 41-49.

Editor
Thank you for the privilege of reviewing your work. Below you will find my comments, instructions from the Spectrum editorial office, and the reviewer comments.

• References: Please double check these and place into the appropriate format.
• Introduction and throughout: Biosafety level is sometimes abbreviated as BLS (line 50) and sometimes as BSL (line 60). Please harmonize throughout the manuscript.
Response: thank you we harmonized it

• Introduction, Line 88: please write as "1960s", as "60s" sounds quite colloquial.
Response: thank you we modify it

• Introduction, Lines 111-119: Please remove this and place it into Materials and Methods, and rewrite in past tense.
Response thank you we modify it

• Introduction, Lines 120-121: Please delete, it is redundant.
Response thank you, we took care of it

• Results: As also mentioned by Reviewer 1, method details are included in the results. Please separate this into the relevant sections.
Response: Thank you, we hope this version results more lean.

Reviewer #1 (Comments for the Author):

Evidence-based recommendations for shower-out practices: assessing virus removal from hair using a surrogate non-enveloped virus

Anastasia Accoti, Maria N.B. Cajimat, Catherine Olal, Krystle Hensley, Joseph Kozlovac, and Dennis A. Bente

This is an extremely relevant piece of work. I'm impressed with all the work, especially using different types of hair is interesting.

However, the presentation of material and methods, results and discussion needs improvement. Do not repeat material and methods in the results section, the results section can be much shorter. Do not repeat introduction and results in the discussion section.
In the discussion section it would be interesting to discuss pH sensitivity of bovine rhinitis B virus.
Foot-and-mouth disease virus is pH sensitive; it falls apart below neutral pH and is almost fully inactivated at pH 5. (Bachrach, H. L., Breese, S. S., Callis, J. J., Hess, W. R., Patty, R. E. 1957.
Inactivation of Foot-and-Mouth disease virus by pH and Temperature changes and by Formaldehyde. Proc. Soc. Exp. Biol. and Med. 95; 147-152).
I could not find such a paper on bovine rhinovirus B, I assumed acid sensitivity is similar but based on the pH of the products it seems that virus can survive better at low pH. Interestingly the pH of TREsemmé is the lowest (suppentary table 1) and inactivates better (figure 1).

Response: Thank you for the useful feedback, please find this topic discussed in Line

The materials and methods section should be written with more clarity and the Q-RT-PCR that is mentioned in the results should also be mentioned in the M&M section.
Response: The Materials and Methods was modified to include this sentence (lines …)

I would assume that from each wig hairs were collected before washing to estimate the virus concentration.
I could not find this in the materials and methods section nor in the results section, but I see results in the supplementary tables. I do not see virus titres though, only detection of virus, then it would be good to discuss the virus titres found after washing in relation to the virus titre applied (which should be mentioned). The application of 0.5 to 0.8 ml to a whole wig is relatively a small amount.
Response: We decided to back titer the virus harvested from the hair that did not get washed and we add this piece of information in the supplementary table 3 and we discusse it in the discussion session L 308-314 adn 369-373.
Moreover, we add the informatin of titer of each initial virus used to expose the heads in Table1 and we add the specific amount of volume in Supplementary table 3.

Please indicate in the discussion that only non-enveloped virus was tested for survival after showering, as the enveloped viruses were already inactivated by Shampoo.
Asnwer: Thank you, we agree with this comment, in fact you can find in L 281 and in L284 the clear specification that the mechanical removal was done with non-envoloped virus

Specific comments:

The ZZ-R (127) cells are described in https://www.cellosaurus.org/CVCL_5418
Response: We obtained the cells directly from FLI thru Dr. Thomas Ksiazek with an MTA and we thank him in the ackwoledgment section.

Abstract:
Line 25 and 281: delete "highly". Contagiousness is not solely a characteristic of the virus, but also of the host density. In endemic settings the R-naught estimates are less than 1.5, which is not highly contagious.
Response: thank you, we accept the comment, and removed the highly.

Introduction:
Line 67: delete "one of the most" (see above not true and it is not a competition). You could stress the economic consequences more.
Response: Thank you, we accept the comment, and removed one of the most.

Line 73 - 75: Please consider deleting this sentence. The support of airborne transmission is mainly based on believes and not on actual data. In fact, de Bouma et al. 2004 showed that calves housed in the same stable did not transmit when kept in in boxes, whereas Orsel et al. 2004 showed that within the same age group transmission occurred when animals were freely roaming. This shows that the

contribution of airborne transmission in cattle is limited. The work of Sorensen et al. 2000 showed that previous results with Gausian spread models could only be reproduced when more than 100 times more virus output was used (the strain in Isle of Wight did only produce 6.2 log virus per day and not 8.6), extreme susceptibility of cattle was entered in the model and stable conditions above seawater were exaggerated. Although the author did not conclude that previous estimates were wrong, the data clearly show that previous estimates were wrong.
Bouma, A., Dekker, A., de Jong, M. C. M. 2004. No foot-and-mouth disease virus transmission between individually housed calves. Vet. Microbiol. 98(1); 29-36.
Orsel, K., Dekker, A., Bouma, A., Stegeman, J. A., de Jong, M. C. M. 2005. Vaccination against foot and mouth disease reduces virus transmission in groups of calves. Vaccine 23(41); 4887-4894.
Sorensen, J. H., Mackay, D. K. J., Jensen, C. O., Donaldson, A. I. 2000. An integrated model to predict the atmospheric spread of foot-and-mouth disease virus. Epidemiology-and-Infection 124(3); 577-590.
Response: Thank you for the comment, we removed the sentence

Line 111-119: End the introduction with a clear objective, e.g. the objective of the current study was the evaluation of virus inactivation by different hair products (shampoo and conditioner) as well as the effect of washing hair on virus survival. The product names will be mentioned in material and methods. Be concise be precise.
Response: thank you for the feedback, we removed the eccessive technical informations about the hair products and adjust the end of the introduction as suggested

Line 120-121: Delete these lines, they can be used in the discussion.
Response: thank you, we removed it

Line 165: Suggestion for section title: Evaluation of titration of bovine rhinitis B Virus in goat tongue cells
Aswer: thank you for this alternative title, we changed it as you suggested

Line 166-167: can be deleted
Response: thank you for this feedback, but we consider this sentence an important intro for the undertsanding of this result section

Line 170: specify the species from which the kidney cells were cultured (primary porcine kidney cell, primary bovine kidney cells or primary ovine kidney cells).
Response: The primary cells were sourced from bovine, the sentence L 159-160 was modified accordingly.

Line 173: after "tongue cells" add (ZZ-R from Friedrich Loeffler Institute, Germany)
Asnwer: Thank you, however since we kept the other intro sentence, we did not repeat the origin of our Goat Tongue cells.

Results:
The whole result section contains to much material and methods and is not easy to read. Please rewrite.

Response: thank you, we realized we add too many technical information, we hope this current version of the results is more lean in matherial and methods information.

Line 200 to 210: check the use of the word "for". Check the English of this part.
Response: Thank you, we correct it

Line 211 to 214: It is not clear how "cosmetology mannequin head" is different from a bald head with a wig.
Response: We tried to clarify in the text more, and we will explaining here, at the beginning of the study, in order to have a more homogene and standardize source of hair, we ordered different types of hair from the same brand of cosmetology mannequin head, that consist in a mannequin head with laready attached hairs. Unfortunately those specific type of mannequin heads, since they are used for cosmetology purpose resulted severly chemical treated, resulting in total cell death. We optimize the system by buying bold mannequin head, buy a single sample size of hair, test it for cytotpoxicity and then proceed to buy the whole amount of hair and depending if it was a wig or extension, hairs were secured on the bold mannequin head.

Line 227: How many hairs are in a strand?
Response: it depends on the type of hair, in order to standardize the amount, we use the cutting surface area (2 $inches^2$), instead of the amount of hair. This information is present in MM.

Line 227: replace "large" with "long", at least I think that is what you mean.
Response: thank you for your comment, no we meant large, since we measure the amount of hair to collect based on surface area.

Line 227: I do not understand the reference to number 2 (Biosafety in Microbiological and Biomedical Laboratories (BMBL) 6th Edition)
Response: It is a formatting issue, it's used to designate "square" inches (surface area). The sentence was modified to include square inches.

Line 228: the hair is not filtered through 200 nm filter, but the eluted material.
Response: Thank you, we correct the mistake

Discussion:
The discussion should not repeat "Introduction", "Materials and Methods" and "Results". Start the discussion with the objective (often the last sentence in the introduction) and then discuss the results.
Response: thank you for the feedback we improved the discussion

Line 288: Replace "direct-contact liquid suspension assay" by "quantitative suspension test", this is the term also used in norm for disinfectant testing, e.g. EN 14675:2015 - Quantitative suspension test for the evaluation of virucidal activity of chemical disinfectants and antiseptics used in the veterinary area (https://microbe-investigations.com/disinfectant-testing/disinfectant-antiviral/en-14675-virucidal-testing-of-veterinary-disinfectants/)
Response: Modified to liquid suspension assay.

Materials and methods:
I see results of Q-RT-PCR but no reference to the method used in materials and methods. Please add a reference.
Response: The methodology was added to Materials and Methods (line) and reference (Kishimoto et al 2017)

Line 443 and 476: Reed and Muench is not a good method. It assumes results that were not observed. Spearman Karber is superior "The time has come for a change: under the most favorable conditions, neither [the Reed-Muench nor the Dragstedt-Behrens] method is as precise as the Spearman-Karber, and often Spearman-Karber is markedly superior.... Except as part of statistical history, both methods should be forgotten."
Myers, L. E., McQuay, L. J., Hollinger, F. B. 1994. Dilution assay statistics. J. Clin. Microbiol. 32(3); 732-739.
Response: We appreciate the comments regarding the statistical methods used for endpoint estimation. We recognize that alternative approaches, such as the Spearman–Karber method, can offer higher theoretical precision under certain conditions. However, the Reed–Muench method remains widely used and accepted in virological and microbiological titration work due to its simplicity, and transparency. In our context, while we acknowledge the theoretical advantages of alternative methods, the differences in practical outcomes are generally minimal for the intended application and do not materially impact the interpretability of results within our validated workflow. For these reasons, we consider Reed–Muench an appropriate and fit-for-purpose method for our work.

Line 541: It is not clear how many hairs were collected. In the results section you state 2 inch (= 5 cm in SI units) of hair was collected. Not only the length, but also the number of hairs might influence the result.
Response: Thank you, however we could not find in L541 the 2 inch, we always had the same lenght of hair that was always 12 inches. For the Minimum Amount of Non-Enveloped Viral Particles Detected on Synthetic Hair experiment we used 6 inches long hair.

Lin 542 - 543: With "2 minutes and a half" do you mean 2.5 minutes? Which would be clearer to the reader.
Response: thank you, yes we meant 2.5, we changed it.

Line 554: did you check daily or only once during that week?
Response: Thank you for your question, we checked for the P1 every other day and for the P2 48 hours after inoculum to confirm infectious status of the positive and then before the end of the seven days.

Figures:
The colour of Design Essential conditioner in figure 1 is different in the graph compared to the legend.
Response: thank you for the catch, we fix figure 1

Supplementary tables and figures:
The difference between supplementary table 3 and 4 is very small. It is strange that

"Braids 100" has a positive result with TREsemmé-Shampoo which is not found in the second passage. Could this be toxic effect on the cells and no virus? TREsemmé inactivated the virus rather well in the suspension test, so it would be logical that it works best in the washing test.
Response: thank you for the catch, unfortunately we copy and paste end of passage 1 under table end of P2 and viceversa, as you can see from the title of the table. We prompty fix it.

References:
The reference list needs a lot of attention. E.g. number 6 should read
Bøtner, A., Belsham, G. J. 2012. Virus survival in slurry: Analysis of the stability of foot-and-mouth disease, classical swine fever, bovine viral diarrhoea and swine influenza viruses. Vet. Microbiol. 157(1-2); 41-49.
Response: thank you, we are using zotero to make the reference and for some reasons some references resulted butchered, we checked all the references and fix them manually.

Reviewer #2 (Comments for the Author):

Well done paper- the experiments and methodologies were well thought out

Response: Thank you very much

Re: Spectrum00387-26R1 (**Evidence-based recommendations for shower-out practices: assessing virus removal from hair using a surrogate non-enveloped virus**)

Dear Dr. Anastasia Accoti:

Your manuscript has been accepted, and I am forwarding it to the ASM production staff for publication. Your paper will first be checked to make sure all elements meet the technical requirements. ASM staff will contact you if anything needs to be revised before copyediting and production can begin. Otherwise, you will be notified when your proofs are ready to be viewed.

Data Availability: ASM policy requires that data be available to the public upon online posting of the article, so please verify all links to sequence records, if present, and make sure that each number retrieves the full record of the data. If a new accession number is not linked or a link is broken, provide production staff with the correct URL for the record. If the accession numbers for new data are not publicly accessible before the expected online posting of the article, publication may be delayed; please contact ASM production staff immediately with the expected release date.

Publication Fees: For information on publication fees and which article types have charges, please visit our website. We have partnered with Copyright Clearance Center (CCC) to collect author charges. If fees apply to your paper, you will receive a message from no-reply@copyright.com with further instructions. For questions related to paying charges through RightsLink, please contact CCC at ASM_Support@copyright.com or toll free at +1-877-622-5543. CCC makes every attempt to respond to all emails within 24 hours.

ASM Membership: Corresponding authors may join or renew ASM membership to obtain discounts on publication fees. Need to upgrade your membership level? Please contact Customer Service at Service@asmusa.org.

Embargo Policy: A press release may be issued as soon as the manuscript is posted on the Spectrum Latest Articles webpage. The corresponding author will receive an email with the subject line "ASM Journals Author Services Notification" when the article is available online.

The ASM Journals program strives for constant improvement in our submission and publication process. Please tell us how we can improve your experience by taking this quick Author Survey.

Thank you for submitting your paper to Spectrum.

Sincerely,
Kessendri Reddy
Editor
Microbiology Spectrum
