# Supplementary material for: Evidence-based recommendations for shower-out practices: assessing virus removal from hair using a surrogate non-enveloped virus
Source: Microbiol Spectr. 2026 Jul 13;14(8):e00387-26. doi: 10.1128/spectrum.00387-26 (PMC13436421; doi:10.1128/spectrum.00387-26)
Supplement: Supplementary file 2 [file spectrum.00387-26-s0001.docx]

**Supplementary**

**Supplementary Figure 1. Hair products and hairstyles**

Figure 1A: List of hair products used for the liquid suspension assay (A–H) and for the mechanical removal of BRBV by showering (A, C, D, E): shampoo—TRESemmé (A), Gojo Spa Bath and Body (B), Ginger Lily Farms Moroccan Argan Oil (C), Design Essentials Natural Almond and Avocado (D); co-wash—Design Essentials Natural Almond and Avocado (E); leave-in conditioners—Design Essentials Natural Almond and Avocado (F), Hair Rules Nourishment (G), and Melanin Multi-Use Softening (H).

Figure 1B: Representative images of the front, side, and back of a mannequin head equipped with either synthetic or human hair, used in the mechanical removal of BRBV by showering. Synthetic hair types include straight, curly/kinky, loose locs, and 45- and 100-braid styles. Human hair types include straight/wavy, curly/kinky, and mohawk-style locs.

**Supplementary Table 1: Hair product characteristics**

| ***Shampoo*** | **pH in Deionized Water** | **SDS** | **Oil** | **Acid** | **Glycerin** | **Surfactant** | **Weight (g) 1:10** | **Weight (g) 1:5** |
| --- | --- | --- | --- | --- | --- | --- | --- | --- |
| GOJO SPA Bath and Body | 5.5 | >1-<5% | No | Citric | >1-<5% | Cocamidopropyl betaine | 1.28 | N/A |
| TREsemmé | 3.5 | ≤25% | No | Ascorbic, citric | Yes | Sodium C12-13 pareth sulfate, cocamidopropyl betaine, cocamide MEA, TEA-dodecylbenzenesulfonate | 1.37 | 1.6 |
| Ginger Lily Farms Morocoo Argan Oil | 4.5 | 0 | Argan oil, vegetable oil | Ascorbic, citric | Yes | Sodium C14-16 olefin sulfonate, cocamidopropyl hydroxysultaine, cocamide MIPA | 1.53 | 1.1 |
| Design Essentials Natural Almond & Avocado | 5 | 0 | Avocado, sweet almond, vegetable | Benzoic | Yes, ethylhexylglycerin | Sodium C14-16 olefin sulfonate, cocamidopropyl hydroxysultaine, cocamidopropyl betaine, laureth-4 | 1.77 | 1.5 |
| ***Co-wash*** |  |  |  |  |  |  |  |  |
| Design Essentials Almond & Avocado | 6 | 0 | Avocado, sweet almond | No | Yes | Laureth-4, laureth-23, behentrimonium chloride | 1.66 | 2.6 |
| ***Leave-In Conditioner*** |  |  |  |  |  |  |  |  |
| Design Essentials Almond & Avocado Detangling | 3.5 | 0 | Vegetable, sweet almond, avocado, jojoba, tea tree leaf | No | Yes | Trideceth-10 | 1.9 | N/A |
| Hair Rules Nourishment | 6 | 0 | Avocado, olive, jojoba | No | Yes | Ppg-5-Ceteth-20 | 0.95 | N/A |
| Melanin Multi-use softening | 4 | 0 | Jojoba, argan, spearmint | Caprylhydroxamic, citric, wheat amino acids | Yes | Behentrimonium Methosulfate (BTMS) | 1.54 | N/A |

The pH of each of the 1:10 hair product solution was determined with fresh deionized water using pH strips (Fisherbrand). SDS, oil, acid, glycerin and surfactant were obtained from the list of ingredients displayed on the product label. The weight of the 1:5 hair products was only determined for the one used in the mechanical removal experiment. Yes = presence, No = absence, N/A = Not Applicable

**Supplementary Table 2: Details of brands of hair and pre-washed conditions and washing techniques**

| **Style** | **Type (S^1^,H^2^)** | **Origin (M^3^,W^4^, E^5^)** | **Length (inches)** | **Strand Amount** | **Attachment** | **Pre-treatment** | **Brand** | **Description** |
| --- | --- | --- | --- | --- | --- | --- | --- | --- |
| **Straight 1A** | S^1^ | W^4^ | 12 | N/A | Mannequin | Dish Washer | Headfix | Headfix 26in-28in Long Hair Mannequin Head Stnthetic Fiber Hairdresser Practice Styling Training, Cosmetology Manikin Doll Head With Clamp Stand |
| **Curly/Kinky 3C** | S^1^ | W^4^ | 12 | ~415 | Stapler | Dish washer | RunM | RunM Short Curly Afro Wig With Bangs for Black Women Kinky Curly Hair Wig Afro Synthetic Full Wigs（Black) |
| **Loose Locs 3B** | S^1^ | E^5^ | 12 | ~150 | Stapler | Dish Washer | GNIMEGIL | Short Braided Wig for Man Afro Bob Black Crochet Twist Hair Dreadlocks Wig Natural Synthetic Wig Layered Breathable Faux Locs Braids |
|  |  |  |  |  |  |  |  |  |
|  |  |  |  |  |  |  |  |  |
| **Braids_1** | S^1^ | E^5^ | 12 | ~45 | Sew and Stapler | Hand washed | Wodun | 12inch Goddess Box Braids Crochet Hair Crochet Box Braids Curly Ends 3X Bohemian Crochet Box Braids Hair Synthetic Crochet Hair Extensions |
| **Braids_2** | S^1^ | E^5^ | 12 | ~100 | Sew and Stapler | Hand washed | Wodun | 12inch Goddess Box Braids Crochet Hair Crochet Box Braids Curly Ends 3X Bohemian Crochet Box Braids Hair Synthetic Crochet Hair Extensions |
| **Straight/Wavy 2A** | H^2^ | E^5^ | 12 | N/A | Stapler | Hand washed | Eayon Hair | Clip In Hair Extension Silky Straight Brazilian Human Hair |
| **Curly/Kinky**  **4A** | H^2^ | E^5^ | 12 | ~225 | Stapler | Hand washed | Urbeauty | 12 Inch Kinky Curly Clip In Hair Extensions for Black Women Human Hair, Urbeauty Curly Hair Extensions Clip in Human Hair, 3C 4A Kinky Curly Hair Clip Ins for Women |
| **Locs**  **4B** | H^2^ | E^5^ | 12 | ~35 | Stapler | Hand washed | DAIXI | 0.24Inch Thickness 8-16 Inch 60 Strands 100% Real Human Hair Dreadlock Extensions for Man/Women Full Head Handmade 0.6cm Thinner Can Be Dyed and Bleached Soft Dreadlocks with Needle and Comb |

Table is showing the different hairstyles defined by the Andrew Walker’s classification code and the hair types used defined as synthetic (S) or human (H). Hair origin was either from mannequin head (M), wig (W) or extension (E). Hair length is expressed in inches, and the number of strands was determined by counting every strand, synthetic straight and human straight/wavy hairs were not counted. Hair was either already on the mannequin head, or the wigs or the extension were either stappled or sew in elastic bands and then stapled. Hairs were always prewashed either with a dishwasher (only water) or by hand. Brand and detailed description of the hair provided by the manufactures is listed.

**Supplementary Table 3: Results of day 0 Bovine Rhinitis B Virus titer of the hair exposure control-no rinse**

| **Day 0 P0** |  |  |  |  |
| --- | --- | --- | --- | --- |
| **Hair type** | **Area of Hair Collection** | | | **Volume of Viral Exposure** |
| ***Synthetic*** | **Front** | **Central** | **Back** |  |
| Straight | N | Y* | N | 500 |
| Curly/Kinky | 3x10 | N | N | 500 |
| Loose-Locs | 3.1x10 | N | N | 500 |
| Braids 45 | 1.4x10 | 1.4x10 | Y* | 500 |
| Braids 100 | 3.1X10^3^ | 1x10^2^ | 1.7x10^2^ | 700 |
| ***Human*** |  |  |  |  |
| Straight/Wavy | 1.4x10 | 4x10 | 4x10 | 800 |
| Curly/Kinky | 3.1x10 | 4x10 | N | 700 |
| Locs | 1x10^2^ | 3.1x10^2^ | 2.4x10^2^ | 700 |

The table is showing the virus titer as determined by TCID50 harvested from the viral exposure control-no rinse the day of the BRBV hair exposure and the volume of virus suspension used to expose each head. The volume is expressed in μL. * Means titer is lower than 1 log_10_. Y = BRBV-infected, N = Not Infected

**Supplementary Table 4: Results of CPE at the end of passage 1 of mechanical removal of non-enveloped virus from synthetic and human hairs by showering**

| **Day 7 P1** | **Hair Treatment** | | | | | | | | | | | | | | | | | |
| --- | --- | --- | --- | --- | --- | --- | --- | --- | --- | --- | --- | --- | --- | --- | --- | --- | --- | --- |
| **Hair type** | **TRESemmé-**Shampoo | | | **Ginger-**Shampoo | | | **De Essentials**-Shampoo | | | **De Essentials-**  Co-Wash | | | **Water** | | | **No Rinse** | | |
| ***Synthetic*** | **F** | **C** | **B** | **F** | **C** | **B** | **F** | **C** | **B** | **F** | **C** | **B** | **F** | **C** | **B** | **F** | **C** | **B** |
| Straight | N | N | N | N | N | N | N | N | N | N | N | N | N | N | N | Y | Y | N |
| Curly/Kinky | N | N | N | N | N | N | N | N | N | N | N | N | N | N | N | Y | Y | N |
| Loose-Locs | N | N | N | N | N | N | N | N | N | N | N | N | N | N | N | Y | Y | N |
| Braids 45 | N | N | N | N | N | N | N | N | N | N | N | N | N | N | N | Y | Y | Y |
| Braids 100 | N | N | N | N | N | N | N | N | N | N | N | N | Y | N | Y | Y | Y | Y |
| ***Human*** |  |  |  |  |  |  |  |  |  |  |  |  |  |  |  |  |  |  |
| Straight/Wavy | N | N | N | N | N | N | N | N | N | N | N | N | N | N | N | Y | Y | Y |
| Curly/Kinky | N | N | N | N | N | N | N | N | N | N | N | N | N | N | N | Y | Y | N |
| Locs | N | N | N | N | N | N | N | N | N | N | N | N | N | N | N | Y | Y | Y |

The table is showing the end of passage 1 of the infection result of the mechanical removal of the FMD surrogate virus, BRBV, during shower-out practice. Every hair style is divided by shampoo treatment and hair strand collection, including the rinse with only water and the hairs that were exposed to virus but not rinse. F: Front, C: Center, B: Back,Y = BRBV-infected, N = Not Infected

**Supplementary Table 5: Results of the end of passage 2 of mechanical removal of non-enveloped virus from synthetic and human hairs by shower**

| **Day 7 P2** | **Hair Treatment** | | | | | | | | | | | | | | | | | |
| --- | --- | --- | --- | --- | --- | --- | --- | --- | --- | --- | --- | --- | --- | --- | --- | --- | --- | --- |
| **Hair type** | **TRESemmé-**Shampoo | | | **Ginger-**Shampoo | | | **De Essentials**-Shampoo | | | **De Essentials-**Co-Wash | | | **Water** | | | **No Rinse** | | |
| ***Synthetic*** | F | C | B | F | C | B | F | C | B | F | C | B | F | C | B | F | C | B |
| Straight | N | N | N | N | N | N | N | N | N | N | N | N | N | N | N | Y | Y | N |
| Curly/Kinky | N | N | N | N | N | N | N | N | N | Y | N | N | N | N | N | Y | Y | Y |
| Loose-Locs | N | N | N | N | N | N | N | N | N | N | N | N | N | N | N | Y | Y | Y |
| Braids 45 | N | N | N | N | N | N | N | N | N | N | N | N | N | N | N | Y | Y | Y |
| Braids 100 | Y | N | N | N | N | N | N | N | N | N | N | N | Y | N | Y | Y | Y | Y |
| ***Human*** |  |  |  |  |  |  |  |  |  |  |  |  |  |  |  |  |  |  |
| Straight/Wavy | N | N | N | N | N | N | N | N | N | N | N | N | N | N | N | Y | Y | Y |
| Curly/Kinky | N | N | N | N | N | N | N | N | N | N | N | N | N | N | N | Y | Y | N |
| Locs | N | N | N | N | N | N | N | N | N | N | N | N | N | N | N | Y | Y | Y |

The table is showing the end of passage 2 of the infection result of the mechanical removal of the FMD surrogate virus, BRBV, during shower-out practice. Every hair style is divided by shampoo treatment and hair strand collection, including the rinse with only water and the hairs that were exposed to virus but not rinse. F: Front, C: Center, B: Back, Y = BRBV-infected, N = Not Infected.
